# Role of antithrombotic therapy in the risk of hematoma recurrence and thromboembolism after chronic subdural hematoma evacuation: a population-based consecutive cohort study

**DOI:** 10.1007/s00701-017-3330-x

**Published:** 2017-09-27

**Authors:** Ida Fornebo, Kristin Sjåvik, Mark Alibeck, Helena Kristiansson, Fredrik Ståhl, Petter Förander, Asgeir Store Jakola, Jiri Bartek

**Affiliations:** 1 Faculty of Health Sciences, University of Oslo, Oslo, Norway; 2 Department of Neurosurgery, University Hospital of North Norway, Tromsö, Norway; 3 Department of Clinical Neuroscience, Karolinska Institutet, and Department of Neurosurgery, Karolinska University Hospital, Stockholm, Sweden; 4 Department of Neurosurgery, St.Olav University Hospital, Trondheim, Norway; 5 Department of Neurosurgery, Sahlgrenska University Hospital, Gothenburg, Sweden; 6 Institute of Neuroscience and Physiology, University of Gothenburg, Sahlgrenska Academy, Gothenburg, Sweden; 7 Department of Neurosurgery, Copenhagen University Hospital Rigshospitalet, Copenhagen, Denmark

**Keywords:** Chronic subdural hematoma, Neurosurgery, Treatment outcomes, Antiplatelet, Anticoagulants, Antithrombotics, Recurrence, Thromboembolism

## Abstract

**Objective:**

To establish the risk of recurrence in patients with chronic subdural hematoma (cSDH) on antithrombotic treatment (AT, i.e., antiplatelets and anticoagulants). Secondary end points were perioperative morbidity and mortality between groups (AT vs. no-AT group) and exploration if timing of resumption of AT treatment (i.e., prophylactic early vs. late resumption) influenced the occurrence of thromboembolism and hematoma recurrence.

**Materials:**

In a population-based consecutive cohort, we conducted a retrospective review of 763 patients undergoing primary burr hole procedures for cSDH between January 1, 2005, and December 31, 2010, at the Karolinska University Hospital, Stockholm, Sweden. Early AT resumption was ≤30 days and late >30 days after the procedure.

**Results:**

A total of 308/763 (40.4%) cSDH patients were on AT treatment at the time of diagnosis. There was no difference in cSDH recurrence within 3 months (11.0% vs. 12.0%, *p* = 0.69) nor was there any difference in perioperative mortality (4.0% vs. 2.0%, *p* = 0.16) between those using AT compared to those who were not. However, perioperative morbidity was more common in the AT group compared to no-AT group (10.7% vs. 5.1%, *p* = 0.003). Comparing early vs. late AT resumption, there was no difference with respect to recurrence (7.0% vs. 13.9%, *p* = 0.08), but more thromboembolism in the late AT resumption group (2.0% vs. 7.0%, *p* < 0.01).

**Conclusion:**

In clinical practice, cSDH patients on AT therapy at the time of diagnosis have similar recurrence rates and mortality compared to those without AT therapy, but with higher morbidity. Early resumption was not associated with more recurrence, but with lower thromboembolic frequency. Early AT resumption seems favorable, and a prospective RCT is needed.

## Introduction

Chronic subdural hematoma (cSDH) is a common neurosurgical condition, with increasing incidence rates among the elderly [1, 2, 6, 7, 13, 16, 25, 34]. In this segment of the population, comorbid conditions are common, and many use antithrombotic medication, that is, antiplatelet and anticoagulant treatment [18]. Due to the comorbidity in this patient population, balancing thromboembolic events against the risk of both acute hemorrhage and recurrence presents a challenge in the management of cSDH patients [14, 20, 22, 23, 27, 31, 32].

AT predisposes to cSDH, and it is common practice to stop treatment and await drug clearance or reverse the AT effect at presentation and certainly before surgical treatment [4, 15, 19]. On the other hand, it is still controversial when to resume AT in cSDH patients after surgery. Early (vs. late) resumption has been reported to predispose to recurrence [4, 12, 27, 31] as well as to protect against it [10, 20]. Further, the risk of thromboembolism when withholding AT therapy is still heavily debated and unsettled [12, 17, 19, 32, 35], adding to the difficulties in the management of this patient category.

The aims of this study were to establish the risk of cSDH recurrence as well as perioperative mortality and morbidity in patients on AT therapy at time of presentation. Further, we intended to examine whether early (≤ 30 days) vs. late (> 30 days) prophylactic resumption of AT therapy affects the risk for recurrence/intracranial bleedings or thromboembolic events in a population-based consecutive setting.

## Methods

### Patient population

All adult (18 years or above) patients treated with burr hole evacuation of primary cSDH between January 1, 2005, and December 31, 2010, at the Department of Neurosurgery at Karolinska University Hospital (Stockholm, Sweden) were identified using the hospital's patient administrative databases and operating room logs. Patients having undergone any other form of intracranial surgery during the last 6 months prior to cSDH intervention and those having cSDH in relation to arachnoid cysts were excluded.

### Swedish health care system

The health care system in Sweden is divided into different geographical regions with compliant referral patterns for intracranial surgery within these regions. A patient with a cSDH in the greater Stockholm region will be referred to and cared for at the Neurosurgical Department at the Karolinska University Hospital. Due to a strict regional referral, the risk of referral bias is practically eliminated, ensuring population-based data.

### Treatment regimens and follow-up

The treatment of a unilateral cSDH consists of a single burr hole, cruciate dural opening and perioperative irrigation. Immobilization and subgaleal suction drainage were used for 24 h postoperatively [33]. Patients were immobilized during drainage and kept in intermediate ward units. Follow-up consisted of an outpatient visit approximately 4–8 weeks after surgery. A follow-up CT scan was performed only if on a clinical indication.

### Study variables

We defined “index operation” as the first surgical procedure on the affected side. Bilateral hematomas were registered as one index operation if both sides were treated as part of the same procedure. A recurrent cSDH was defined as a same-sided cSDH recurrence treated with surgery within 6 months of the index operation. In the cases where a one-sided index operation was followed by a bilateral recurrent procedure (i.e., one recurrent side and one untreated side), the patient was still registered as having only one index operation and one reoperation.

Patient and treatment characteristics were retrospectively retrieved from medical records. The Charlson Comorbidity Index was used to assess comorbidity [11]. The Landriel Ibanez classification was used to evaluate complications within 30 days postoperatively [24]. Patients dead within 30 days of surgery without resumption of AT contributed to the no resumption group.

### End points

The primary end point was risk of recurrence in AT users. Secondary end points were perioperative morbidity and mortality between groups (i.e., AT vs. no-AT group). Finally, we also examined whether the timing of resumption of AT treatment, i.e., early vs. late prophylactic resumption, where the latter group consists of event-based, late prophylactic or no resumption, later referred to as simply “late” resumption, influenced the occurrence of thromboembolism and hematoma recurrence. Early resumption was ≤ 30 days and late > 30 days after the procedure, based on our institutional clinical practice. Recurrence was defined as any same-sided recurrence in need of repeated surgery within 6 months. Postoperative hemorrhage was defined as any significant acute bleeding within 30 days that qualified for registration as a complication, that is, that it affected postoperative care.

### Statistics

All analyses were done with SPSS, version 24.0 (Chicago, IL, USA). The statistical significance level was set as *p* ≤ 0.05. All tests were two-sided. Central tendencies are presented as means ± SD or medians (interquartile range) in case of skewed distribution. Comparisons of groups when the dependent variable was continuous and there was a dichotomous grouping variable were analyzed with an independent sample t-test when data were normally distributed and with the Mann-Whitney U test when data were skewed. Categorical data were analyzed with Pearson’s chi-square test. Comparisons of means between groups were analyzed using independent samples Student’s t-test.

### Ethics

The study was approved by the Stockholm regional ethical review board in Sweden (EPN 2013/591–31/1).

## Results

In total, 763 patients were included. Among these, 308 patients (40.4%) used AT therapy at the time of diagnosis. These patients differed significantly from non-users in several aspects: more males (71.8% versus 65.1%, *p* = 0.05) and more often CCI > 1 (48.7% versus 26.2%, *p* < 0.001) and GCS < 13 (12.6% versus 5.9%, *p* = 0.001). Patients on AT therapy were also older (78 ± 9 years versus 71 ± 13 years, p < 0.001). Densities of the cSDH, midline shift and bilateral presentation were not different between groups. Interestingly, the proportion having recurrences was not different between groups (11.7% in the group having AT therapy versus 10.8% in patients using no AT, *p* = 0.69). Also, perioperative mortality was not different with 4.2% in the AT therapy group compared to 2.4% in those without AT therapy (*p* = 0.16). However, less morbidity was observed in the no AT group (10.7% vs. 5.1%, *p* = 0.003). This was also significant when only moderate to severe complications as defined by Ibanez grade ≥2 were analyzed (6.8% vs. 3.5%, *p* = 0.04).

Finally, we looked at the AT therapy group with respect to early (30 days or less) events, that is, a thromboembolic event or cSDH recurrence or acute intracranial hemorrhage. See Table 1 for more data. Overall, there was a higher frequency of recurrence/bleeding (8.4%) than of thromboembolism (4.9%) in this period, which was later defined as early resumption versus late resumption.

**Table 1:** Recurrence/bleeding and thromboembolic events within 30 days in users of antithrombotic medication at the time of diagnosis (*n* = 308)

**Events**	**0–3 days**	**4–14 days**	**15–30 days**	**Early (0–30 days)**
**Recurrence/intracranial bleeding (%)**	11 (3.6%)	6 (1.9%)	9 (2.9%)	26 (8.4%)
**Thromboembolic events (%)**	4 (1.3%)	5 (1.6%)	6 (1.9%)	15 (4.9%)

### Early vs. late reinsertion of AT

In the baseline characteristics comparison of early (0–30 days) versus late (> 30 days) resumption of AT treatment, no significant differences were found; see Table 2 for more details. The difference regarding length of withholding the AT preoperatively (days, IQR) was 1 (1–2) in the early resumption group vs. 1 (1–4) in the late resumption group (*p* = 0.04). Also, time to prophylactic AT resumption was (days, IQR) 15 (7–30) in the early resumption group vs. 66 (43–116) in the late resumption group (*p* < 0.001). Besides the AT indications noted in Table 2, there were those receiving AT as a measure of primary prevention or other indications such as a pacemaker, cancer including leukemias, APC resistance and polycethemia vera (*n* = 12 in the early and *n* = 8 in the late resumption group). We had no patients on new oral anticoagulants (NOACs). Bridging consists of a higher dose of LMWH therapy than just perioperative thromboembolism prophylaxis.

**Table 2:** Baseline characteristics comparing patients with early (0–30 days) versus late (> 30 days) resumption of antithrombotic therapy (early, *n* = 100 versus late, *n* = 201), 6 missing because of incomplete data

	**Early resumption (** ***N*** ** = 100)**	**Late resumption (** ***N*** **= 202)**	***P*** **-value**
**Age, years ± SD**	78.1 (9.4)	78.6 (9.4)	0.70
**Female, n (%)**	28 (28.0)	59 (29.2)	0.83
**Charlson comorbidity index >1, n (%)**	55 (55.0)	93 (46.0)	0.14
**Level of independence**			0.85
**Independent, n (%)**	69 (70.4)	137 (67.8)	
**Homecare/nursing home, n (%)**	29 (29.6)	65 (32.2)	
**Missing, n = 2**			
**Glasgow Coma Scale (GCS), n (%)**			0.42
**GCS 3–8**	6 (6.2)	6 (3.0)	
**GCS 9–12**	9 (9.3)	17 (8.6)	
**GCS 13–15**	82 (84.5)	175 (88.4)	
**Missing, n = 7**			
**Hematoma density, n (%)**			0.18
**Hypodense**	29 (30.2)	40 (20.2)	
**Mixed density**	40 (41.7)	106 (53.5)	
**Isodense**	24 (25.0)	48 (24.2)	
**Hyperdense**	3 (3.1)	4 (2.0)	
**Missing, n = 8**			
**Midline shift, mm ± SD**	7.5 (4.2)	7.6 (4.4)	0.94
**Missing, n = 7**			
**Platelet inhibitor, n (%)**	62 (62.0)	131 (64.9)	0.63
**Aspirin**	59 (59.0)	124 (61.4)	
**Clopidogrel**	4 (4.0)	8 (4.0)	
**Dipyridamole**	7 (7.0)	8 (4.0)	
**Anticoagulant, n (%)**	40 (40.0)	73 (36.1)	0.51
**Warfarin**	37 (37.0)	72 (35.6)	
**LMWH**	3 (3.0)	1 (0.5)	
**Combined platelet inhibitor + anticoagulant, n (%)**	2 (2.0)	2 (1.0)	0.47
**Main indication(s) of AT therapy, n (%)**			
	28 (28.0)	52 (25.9)	0.69
**TIA/stroke**	27 (27.0)	46 (22.8)	0.42
**Coronary heart disease**	8 (8.0)	9 (4.5)	0.21
**Mechanical valve**	6 (6.0)	11 (5.4)	0.84
**Peripheral vascular disease**	40 (40.0)	86 (42.8)	0.64
**Atrial fibrillation**	3 (3.0)	4 (2.0)	0.58
**Venous thromboembolism**	22 (22.0)	32 (15.8)	0.20
**Multiple indications**			
**Reversal of AT effect, n (%)**	37 (37.0)	77 (38.1)	0.85
**Bridging AT therapy, n (%)**	23 (23.0)	32 (15.8)	0.13
**Last INR > 1.5 preop, n (%)**	2 (2.1)	7 (3.7)	0.46
**Missing,** ***n*** **= 14**			
**General anesthesia, n (%)**	3 (3.0)	5 (2.5)	0.79
**Bilateral surgery, n (%)**	15 (15.5)	34 (17.2)	0.71
**Missing, n = 7**			

Abbreviations: LMWH = low molecular weight heparin, TIA = transient ischemic attack

In the outcome comparison, early (0–30 days) versus late (> 30 days) resumption, there was no significant difference in the reoperation frequency (7% early vs. 13.9% late, *p* = 0.08), but less thromboembolism in the early resumption group (2% early vs. 11% late, *p* < 0.01). No significance was observed regarding complications (7% early vs. 14.4% late, *p* = 0.06) and moderate to severe complications according to the Ibanez grading scale (3% early vs. 7.9% late, *p* = 0.10) and 30-day mortality (1.0% early vs. 5.5% late, p = 0.06). See Table 3 for more details.

**Table 3:** Outcome variables (*n* = 302, 6 missing and not included because of incomplete data with respect to the timing of AT resumption)

	**Early resumption (N = 100)**	**Late resumption (N = 202)**	***P*** **value**
**Reoperation, n (%)**	7 (7.0)	28 (13.9)	0.08
**Time to reoperation or bleeding event, n(%)**			0.40
**< 1 month**	5 (71.4)	13 (46.4)	
**1–2 months**	2 (28.6)	11 (39.3)	
**2–6 months**	0	4 (14.3)	
**Thromboembolic event, n (%)**	2 (2.0)	22 (11.0)	**<0.01**
**Myocardial**	1 (1.0)	6 (3.0)	
**TIA**	0 (0.0)	7 (3.5)	
**Stroke**	0 (0.0)	5 (2.5)	
**Pulmonary embolism**	0 (0.0)	3 (1.5)	
**Deep vein thrombosis**	1 (1.0)	3 (1.5)	
**Peripheral/other**	0	1 (0.5)	
**Time to thromboembolism, n (%)**			0.52
**< 1 month**	1 (50.0)	14 (63.6)	
**1–2 months**	1 (50.0)	4 (18.2)	
**2–6 months**	0	4 (18.2)	
**Event-based resumption within 30 days**	NA	10 (20.2)	NA
**Complication, n (%)**	7 (7.0)	29 (14.4)	0.06
**Landriel Ibanez grade ≥ 2, n (%)**	3 (3.0)	16 (7.9)	0.10
**Mortality**			
**30-day mortality, n (%)**	1 (1.0)	11 (5.5)	0.06
**90-day mortality, n (%)**	4 (4.0)	14 (7.0)	0.31

### Anticoagulant vs. antiplatelet subgroup analysis

In the exploratory subgroup analyses we wanted to compare the risk for cSDH recurrence in need of reoperation or any significant bleeding event within 30 days between users of antiplatelets and users of anticoagulants. After excluding the four patients using both (*n* = 304), we found no significant difference (6.2% for antiplatelets and 11.7% for anticoagulants, *p* = 0.09). There was also no significant difference when analyzing early versus late resumption in pure subgroups of antiplatelet (*p* = 0.25) and anticoagulant use (*p* = 0.45, data not shown).

In addition, since clinicians most often will face the indication of an anticoagulant for atrial fibrillation, we analyzed this separately (*n* = 78). Within 30 days, recurrence in need of reoperation and significant intracranial bleeding events occurred in 14.1% (*n* = 11) and thromboembolic events occurred in 6.4% (*n* = 5). No patients with atrial fibrillation who received early prophylactic resumption of anticoagulations (*n* = 27) had a thromboembolic event compared to six (11.8%) with an event among those with late resumption (*p* = 0.06). Concerning recurrence in need of reoperation or significant bleeding events within 30 days, 13.7% (*n* = 7) with event-based or late resumption experienced an event compared to 11.1% (*n* = 3) in patients early prophylactic resumption.

## Discussion

In this population-based study of patients with cSDH homogeneously treated with burr-hole evacuation and closed drainage, we did not find any significant difference in recurrence rate in the AT vs. no-AT group, nor did we find any significance with respect to perioperative mortality between groups. However, there was a higher perioperative morbidity in the AT group. Early resumption is not associated with more recurrence, but lower thromboembolic frequency. Thus, early AT reinsertion (defined as within 30 days or less) seems beneficial.

### AT versus no-AT group

Literature shows little to no evidence when it comes to correlating the risk of cSDH recurrence to AT therapy in general [3, 5, 9, 20], as was the case in our series, with no association between recurrence and AT therapy found (*p* = 0.69). Also, perioperative mortality was not significantly different with 4% in the AT therapy group compared to 2% in those without AT therapy (*p* = 0.16). However, significantly less morbidity was observed in the no AT group (10.7% vs. 5.1%, *p* = 0.003). This was also significant when only moderate to severe complications as defined by Ibanez grade ≥ 2 were analyzed (6.8% vs. 3.5%, *p* = 0.04), probably as a surrogate measure for patients with more comorbidity use AT to a greater extent than those without comorbidities. This is also reflected by our data with AT users being older, with more comorbidities and in addition in a worse clinical condition (lower GCS score) than patients without AT.

Interestingly, in the AT group, looking at the thromboembolic event and cSDH recurrence within 30 days, the risk of recurrence/acute hemorrhage was more common than thromboembolic events (8.4% vs. 4.9%), a finding corroborated by Guha et al. [20], demonstrating a postoperative risk of 19% for recurrence/acute hemorrhage vs. 2.6% for thromboembolism in their AT group. This is the group with the presumed highest pretest likelihood for experiencing a thromboembolic event. Consequently, it should come as no surprise that ATs are withheld as long as 30 days in this patient category, as was the case in our study (i.e., the policy at our institution), separating early vs. late reinsertion of AT.

### Early versus late resumption of AT

Bleeding and thromboembolism risk in the postoperative period: the question when to resume AT after cSDH evacuation is a frequent one in the neurosurgical daily practice, with a recent study revealing that most surgeons relied on their “own intuition and past experiences” when making this decision [21]. Clearly, the question of optimal resumption has not yet been answered, although there is no doubt that timely resumption saves lives, as it has been shown that, i.e., in patients with a history of CAD, ASA reduces mortality by >20% [8]. Very few studies have tried to address the question of timely resumption, with evidence suggesting no difference with respect to the recurrence rate between early and late resumption of antiplatelet therapy [29], with others demonstrating that even a combination of antiplatelet + anticoagulant treatment resumption can be made safely at an early stage [20], while still others find the current data inconclusive [3]. The conflicting evidence could possibly be explained by the fact that the studies might have been underpowered considering their relatively small sample size of patients on AT therapy/all included in the study: *n* = 231/479 [20], *n* = 58/448 [29] and *n* = 104/239 [3]. Further, none of these reports assessed the risk of thromboembolism with respect to timely resumption.

Although no difference in cSDH recurrence between groups was observed in our material, an increased thromboembolic frequency in the late group was present. This finding is in line with the results from a recent study demonstrating increased thromboembolic frequency (11.5% vs. 6.4%) in the late resumption group [23]. Also, reoperation was necessary at almost double frequency (13.9% of patients with late resumption versus 7.0% in those with early AT resumption); it did not reach statistical significance. A similar situation was seen concerning perioperative mortality (late resumption group 5.5% versus 1.0% in the early resumption group). While this does not meet the statistical criteria for significance, this may be related to the sample size. In an observational study, it is also possible that this is affected by confounding by indication (i.e., severe comorbid or even terminal patients or patients with bleeding events will not get early resumption). The finding of the beneficial outcome in the early resumption group is nevertheless encouraging, indicating that early resumption should be the norm unless clear contraindications are present.

In general, the lack of evidence in this matter is not all that surprising. The number of variables, including the individual risk profile, potency of the drug in question, dosage and time of resumption, makes it challenging to perform an RCT, as pointed out in a recent review [10]. The review suggested prospective registries as a solution to obtaining more data to guide decision making on this matter. Until such data become available, the authors conclude that each patient should be assessed individually based not only on the surgeons’ opinion concerning the surgical risks (i.e., risk of recurrence), but also based on current evidence concerning the risk of thromboembolism without anticoagulation using the CHA_2_DS_2_-VASc scoring system [26, 30]. In fact, we think it is possible to use a scoring system like this with a predefined range where clinical equipoise exists (i.e., not a mechanical valve) to be eligible for randomization to early versus late resumption in an otherwise pragmatic manner, thus making an RCT on this matter conceivable.

### Anticoagulants versus antiplatelet subgroup analysis

Although no association between cSDH recurrence and AT therapy was found in the literature or in our series, some reports demonstrate a certain correlation when anticoagulation treatment is analyzed as a subgroup [12, 28], whereas subgroup analyses on antiplatelet treatment show no correlation [12, 28]. These reports are similar to our own, showing a trend toward higher recurrence rates for anticoagulant vs. antiplatelet use (11.7% for anticoagulants and 6.2% for antiplatelets), although this was not significant.

### Strengths and limitations

Limitations inherent to retrospective assessment are present in this study (as in the others), although our population-based setting makes it less prone to selection bias. Another limitation is the lack of long-term follow-up data, although the data on cSDH recurrence and postoperative thromboembolism frequency remain indisputable, which in combination with a very low rate of missing data provides reliable data on the risk/benefit of early vs. late resumption of AT therapy in operated cSDH patients.

Further, the validity of the study is limited by the surgeons often subjective decision on when to resume AT therapy; nevertheless, at our institution, the policy has been to resume AT therapy no earlier than after 3–4 weeks unless a strong indication for early AT resumption was present. Resumption may still be confounded by indication since AT may be withheld in presumed high-risk patients for reasons we cannot control in retrospect despite having comparable baseline characteristics. Although early reinsertion of AT seems beneficial, we believe that an RCT, as suggested above, is conceivable and the only way to provide conclusive evidence on this matter because of the potential abovementioned limitations.

## Conclusion

cSDH patients on AT therapy equal those without AT therapy in terms of recurrence rate and perioperative mortality, but have an increased perioperative morbidity. Early resumption is not associated with increased risk of reoperation, but lower thromboembolic frequency. Early resumption of AT therapy seems beneficial, although an RCT is needed to determine the superiority of early resumption in cSDH patients.
